# Supplementary figures and images for: Biallelic structural variations within FGF12 detected by long-read sequencing in epilepsy
Source: Life Sci Alliance. 2023 Jun 7;6(8):e202302025. doi: 10.26508/lsa.202302025 (PMC10248215; doi:10.26508/lsa.202302025)

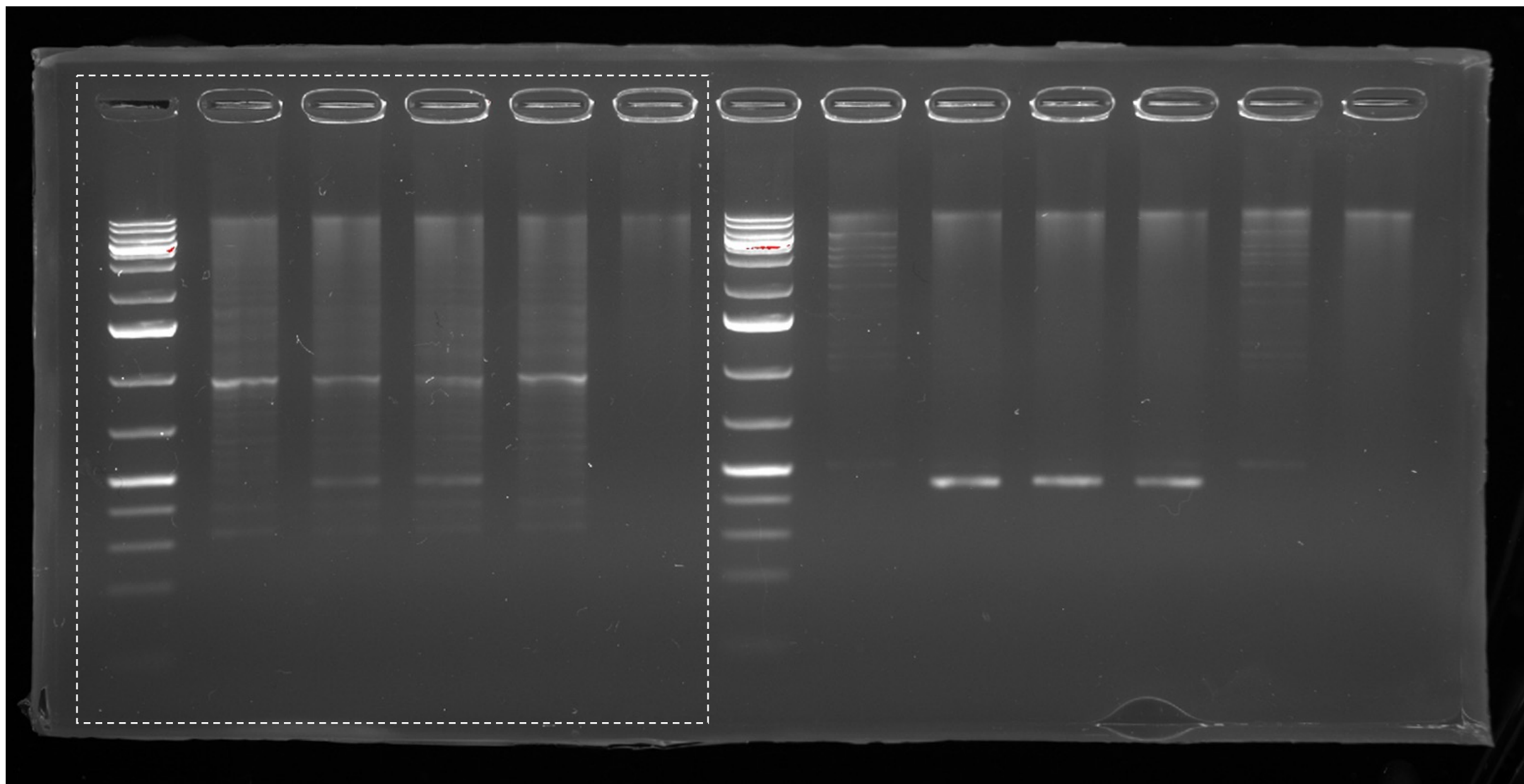

Supplement: Supplementary file 3 [file LSA-2023-02025_SdataFS1.pdf]

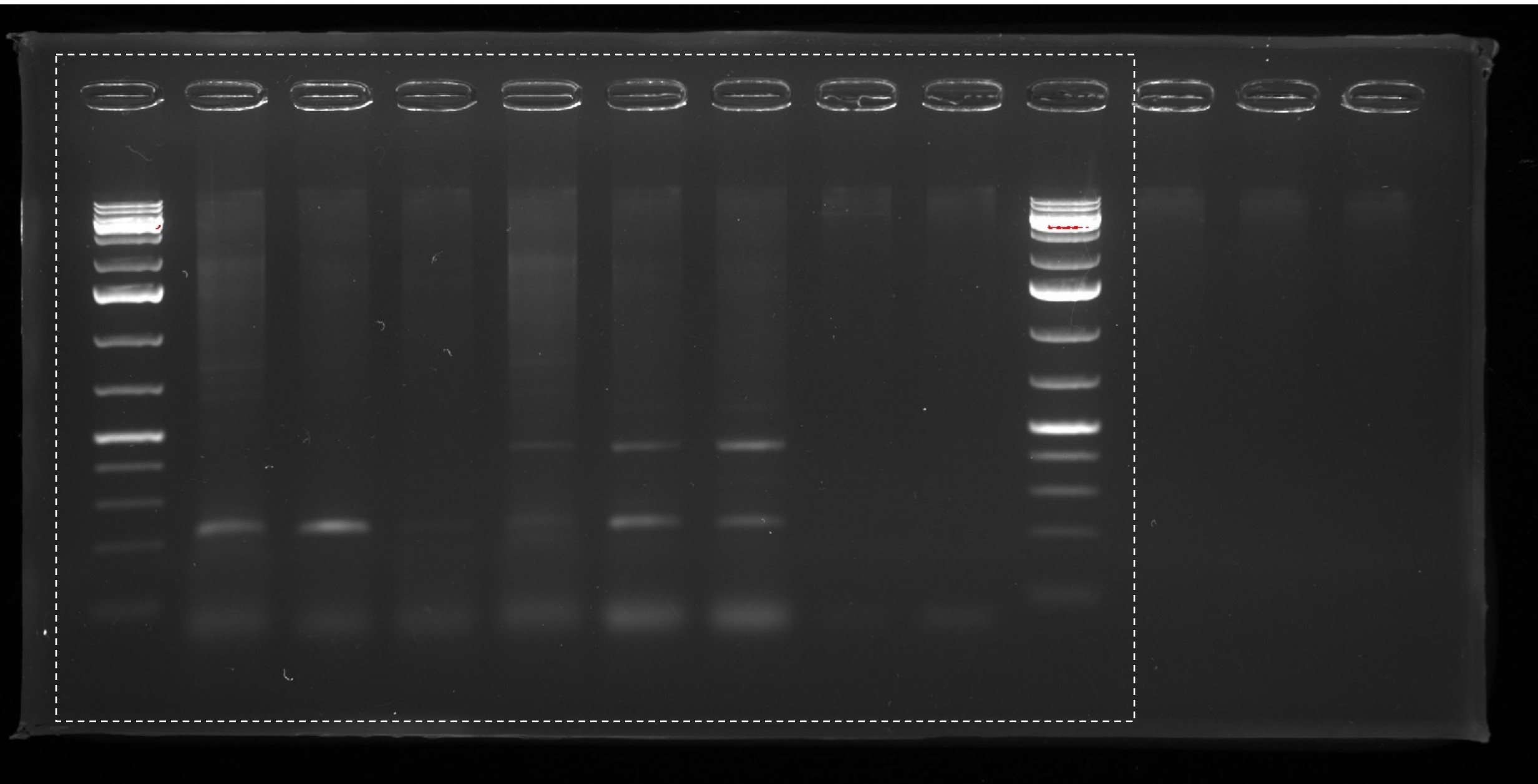

Supplement: Supplementary file 7 [file LSA-2023-02025_SdataF5.pdf]
